# Efficacy of commercially available biological agents for the topical treatment of cervical intraepithelial neoplasia: a systematic review

**DOI:** 10.1186/s13643-019-1050-4

**Published:** 2019-06-07

**Authors:** Alex Baleka Mutombo, Cindy Simoens, Rahma Tozin, Johannes Bogers, Jean-Pierre Van Geertruyden, Yves Jacquemyn

**Affiliations:** 1 Department of Obstetrics and Gynecology, Kinshasa University Hospital, University of Kinshasa, PO Box 236, Kinshasa XI, Democratic Republic of the Congo; 2 Applied Molecular Biology Research Group (AMBIOR), Faculty of Medicine and Health Sciences, Laboratory of Cell Biology & Histology, Universiteitsplein 1, 2610 Wilrijk, Belgium; 3 Global Health Institute, Faculty of Medicine & Health Sciences, University of Antwerp, Doornstraat 331, 2610 Wilrijk, Belgium; 4 Department of Obstetrics and Gynaecology, UZA Antwerp University Hospital and ASTARC, Antwerp University UA, Wilrijkstraat 10, 2650 Edegem, Belgium

**Keywords:** Topical therapy, Cervical intraepithelial neoplasia, Systematic review

## Abstract

**Background:**

Cervical cancer is a major public health issue in the world, especially in developing countries. It can be prevented through vaccination against HPV (primary prevention) and through screening and treatment of cervical intraepithelial neoplasia (CIN) (secondary prevention). Surgical methods for treatment of CIN are linked to complications such as bleeding and adverse pregnancy outcomes. Furthermore, these methods are not generally available in resource-poor settings. Therefore, topical agents for local application on the cervix have been used since decades to overpass complications and limitations of the surgical methods.

**Aims:**

Review of the literature on the efficacy of commercially available biological agents used for topical treatment of cervical intraepithelial neoplasia (CIN).

**Methods:**

A systematic search through PubMed and the Cochrane database was performed up to December 2017, using the medical subheadings (MesH) for topical agent, treatment, and cervical intraepithelial neoplasia. Appropriate inclusion/exclusion criteria have been used for the selection of eligible clinical studies. Clinical studies containing a minimum of 20 women, aged 18–50 with a diagnosis of CIN 1–3, and at least a 4 weeks follow-up after the end of the topical treatment were included.

**Results:**

The initial electronic database search resulted in a total of 849 articles. After screening titles and abstracts, 62 articles were selected as potential studies. Of these, six articles were included in the review after reading the full text: two were on 5-FluoroUracil, two on trans retinoic acid, one on Imiquimod, and one on Cidofovir. The reported regression/remission rates for CIN differed among studies. In CIN2 patients, the overall remission rate ranged between 43 and 93% for the active agents.

**Conclusion:**

Among the topical agents studied, 5-FluoroUracil showed good remission rates above 80%. Varying results seen in this review is due to the differences in quality of the design between studies. Large-scale and less biaised studies are needed to elucidate the true efficacy and safety of topical agents in the treatment of CIN.

**Electronic supplementary material:**

The online version of this article (10.1186/s13643-019-1050-4) contains supplementary material, which is available to authorized users.

## Introduction

Cervical cancer (CC) develops through several, easily detectable precancerous stages. Detection and treatment of the precancerous stages constitutes the mainstay of secondary cervical cancer prevention. Despite the proven efficacy of this secondary prevention, CC continues to be a major public health problem among women globally. Each year, over 500.000 women are diagnosed with CC worldwide, with 266,000 related deaths; making it the fourth most common cancer among women [1].

The precursor lesion for CC is called cervical intraepithelial neoplasia (CIN) or dysplasia. Agreement between the cytological Bethesda system classification and a histological result is as follows: low-grade squamous intraepithelial lesion (LSIL) in The Bethesda System (TBS) is encompassing human papillomavirus (HPV)/mild dysplasia (CIN1), while high-grade squamous intraepithelial lesions (HSIL) is encompassing moderate and severe dysplasia (CIN2 and CIN3) [2]. CIN is induced by a sexually transmitted infection with an oncogenic strain of the HPV [3, 4]. When persisting, it can progress to cervical cancer over a period of 7 to 20 years [5]. With cytological screening, classified by TBS, low- or high-grade lesions can be detected, and used for referral for further histological diagnosis. Histological examination of whole tissues allows several other features to be examined: differentiation, maturation and stratification of cells, and nuclear abnormalities. Nuclear abnormalities such as enlarged nuclei, increased nuclear-cytoplasmic ratio, increased intensity of nuclear staining (hyperchromasia), nuclear polymorphism, and variation in nuclear size (anisokaryosis) are assessed when a diagnosis of CIN is being made. The proportion of the thickness of the epithelium showing mature and differentiated cells is used for grading CIN. According to the height of affected cells within the stratified epithelium, dysplasia is classified as mild (CIN1), moderate (CIN2), or severe (CIN3). Cervical cancer can be prevented through HPV vaccination (primary prevention) and through screening and treatment for CIN and early invasive cervical cancer (secondary prevention).

Until a few years ago, the only method of screening for cervical cancer was the Papanicolaou (“Pap”) smear or cytology. In high-income countries, where Pap smears have been used for population-based screening for over three decades, there has been a major reduction in morbidity and mortality from cervical cancer [6]. However, population-based cytology screening in low- and middle-income countries is often unsuccessful because the financial investments to establish and maintain the necessary level of health infrastructure, including laboratory and skilled human resources, are not available or not sufficient in many settings. Newer methods have been developed for cervical cancer screening: molecular HPV screening tests and visual inspection with acetic acid. Molecular HPV testing methods are based on the detection of deoxyribonucleic acid (DNA)/ribonucleic acid (RNA) from high-risk HPV types in vaginal and/or cervical samples. Visual inspection with acetic acid (VIA) is a method for detecting early cell changes that are visible when using a speculum to inspect the cervix with the naked eye after applying dilute (3–5%) acetic acid to it.

When a precursor lesion of CC is detected during screening, effective treatment and follow-up is necessary. The current surgical treatment options for CIN fall into two main categories: procedures that either ablate the abnormal tissue or procedures that excise the area of abnormality. The most common ablative techniques for the treatment of CIN include cryotherapy, laser ablation, and thermocoagulation. The excisional procedures include cold knife conization, laser cone excision, and loop electrosurgical excision procedure (LEEP). Controlled trials show 80–90% success rates in the treatment of CIN, regardless of treatment modality used [7–9]. In the vast majority of cases, treatments used for CIN are mainly excisional procedures. Possible complications of surgical treatment modalities include cervical stenosis, alteration of the cervical mucus, and removal or destruction of the collagen matrix of the cervical stroma. This can impact future pregnancies with an increased risk for preterm delivery and its associated neonatal mortality [10]. Furthermore, surgical procedures may present major difficulties in low- and middle-income countries as electrical current or a continuous supply of medical gasses (carbon dioxide or nitrogen) is needed [11]. Non-surgical topical CIN therapies do not yield these complications and limitations. These therapies retard tumor growth and stimulate the immune response to viruses, leading to the regression of CIN lesions [12]. Table 1 alphabetically lists the nine commercially available biological agents and their mechanism of action used for topical treatment. There are some other agents that have been experimented in the local treatment of CIN: betulinic acid [23], belonging to the class of organic compounds known as triterpenoids; glycyrrhizinic acid [24], a triterpene glycoside compound extracted from the root of the licorice plant *Glycyrrhiza glabra*; and Praneem [25], a polyherbal formulation that has successfully completed phase II efficacy study for the treatment of abnormal vaginal discharge due to reproductive tract infections that act as co-factors for HPV persistence.

**Table 1:** Common biological agents used for topical treatment of CIN

	Agent	Biological activity
1	5-fluorouracil	5-Fluorouracil is a pyrimidine analogue (cytotoxic agent) used in the treatment of cancer. 5-FU acts through inhibition of the target enzyme thymidylate synthase (TS) by the 5-FU metabolite, FdUMP, and Incorporation of this metabolite into DNA, resulting in inhibition of DNA synthesis and function. It stops the cells making and repairing DNA. Topical 5-FU appears to be an effective medical therapy for CIN [13, 14]
2	Beta-glucan	Beta glucans are sugars that are found in the cell walls of bacteria, fungi, yeasts, algae, lichens, and plants, such as oats and barley. They are sometimes used as medicine. They might stimulate the immune system by increasing chemicals which prevent infections. They mediate their antitumour activity by activation or augmentation of the host’s immune system, via activation of leukocytes and production of inflammatory cytokines. Beta-glucan has been shown to increase the spontaneous regression rate of low-grade cytologic abnormalities as well as cervical findings [15].
3	Cidofovir 2%	The acyclic nucleoside phosphonate cidofovir has proved efficacious in the treatment of different clinical manifestations of HPV-induced epithelial cell proliferation. As an antiviral drug, it is incorporated in viral DNA and preferentially inhibits DNA, reduce capacity of HPV positive cells to repair DNA damage. Cidofovir 1% gel is able to inhibit partially or completely cervical dysplasia lesions [16, 17].
4	Curcumin	Curcumin (diferuloylmethane), a yellow substance from the root of the plant *Curcuma longa* Linn., has been demonstrated to inhibit the transcription of HPV16 E6/E7 proteins as early as six hours after treatment and restores the expression of tumor suppressor proteins p53, retinoblastoma protein, and PTPN13.
5	Imiquimod 5%	Imiquimod is an immunomodulator with antiviral and anti-tumor effects. It is a toll-like receptor 7 agonist and induces up regulation of interferon and activation of dendritic cells. Imiquimod (5% cream) has been shown to be safe and effective in the treatment of genital warts caused by low-risk HPV infections. The mechanism for the eradication of genital verrucous lesions with imiquimod may involve the induction of both innate and cellular immunity. Antiviral activity may be stimulated through the induction of cytokines, such as interferon-a (IFN-a), tumor necrosis factor-a (TNF-a), and interleukins (ILs) [18, 19].
6	Interferon alpha and beta	Interferons (IFNs) are a family of glycoproteins and are natural body defenses against viral infections and play important roles in combating tumors and regulating immunity. IFNs perform their effects through binding to cell surface receptors and activating members of the JAK kinase family.
		The antitumor effects result from direct action on the proliferation or antigenic composition of tumor cells, or from the effect of modulation on immune effector cell populations with tumor cell specificities.
		In addition to this, they can have indirect effects, such as modulation of the immune response and inhibition of tumor angiogenesis.Some studies have shown good results from the use of IFN-β for treating CIN cases [20, 21].
7	Trans-retinoic acid	Retinoids are essential for cell growth, differentiation, and cell death. Various retinoids have been shown to inhibit cellular proliferation in cervical cancer cells in several studies.
		All-trans retinoic acid (atRA) is an active metabolite of vitamin A under the family retinoid. Retinoids, through their cognate nuclear receptors, exert potent effects on cell growth, differentiation and apoptosis.
		Retinoic acid either decreases or increases EGF-stimulated growth and EGF-R expression depending on the cell line and culture conditions. HPV-containing cell lines overexpress EGF-R and are more sensitive to retinoïds than normal cells. Increased sensitivity of HPV-containing cells may explain the reversal of premalignant lesions and dysplasias of the cervix by retinoic acid [22]
8	Trichloroacetic acid	Trichloroacetic acid is an analogue of acetic acid in which the three hydrogen atoms of the methyl group have all been replaced by chlorine atoms. It is a chemically destructive acid that burns, cauterizes and erodes the skin and mucosa, resulting in the physical destruction of warty tissue through protein coagulation. The destructive nature of the product frequently extends beyond the superficial wart to encompass the underlying viral infection.

This review is meant to evaluate the efficacy of these non-surgical commercially available topical therapies used to treat CIN lesions.

## Methods

### Inclusion and exclusion criteria

Reports of clinical trials assessing the effect of topical treatment were included, regardless of the publication language, and containing a minimum of 20 women, with the following inclusion criteria: CIN 1 to 3, aged 18–50 and at least a 4-week follow-up after the end of topical treatment. The diagnostic method and the post-treatment assessment were by cytology or histology. Publications were excluded if CIN was not well-documented, the disease studied was a cancer or an intraepithelial neoplasia other than cervical, when surgical therapy alone or in combination with a topical agent was used, or the recipe or drug was not used topically. Studies not using commercially available preparations were also excluded.

### Outcomes

The main outcome was the efficacy of the topical drug administered. Efficacy is expressed in terms of regression of the CIN lesion on post-treatment colposcopy or histology examination.

Regression is defined as improvement from CIN3 to CIN2 or CIN1 or normal, from CIN2 to CIN1 or normal, and from CIN1 to normal. Regression can be partial or complete. Complete regression, also called remission, is defined as a total disappearance of the CIN lesion or improvement from any grade of CIN (CIN 1, CIN 2, or CIN 3) to no CIN.

### Search strategy and selection of studies

Bibliographic databases (PubMed and Cochrane database) were searched up to December 2017 using a predefined search strategy (see below). In addition, the authors screened the reference lists of identified articles. The search strategy, based on a combination of relevant medical subheadings (MeSH), text words and word variants for topical, therapy, cervix, and neoplasia were utilized. The PubMed database was searched using the following key words: ((“Topical” OR “local”) AND therapy) AND ((“CIN” OR “cervical intraepithelial neoplasia” OR “SIL” OR “ squamous intraepithelial lesion” OR “neopl*” OR “dyspl*”) AND (“cervix” OR “cervical”)) NOT animals. The Cochrane database as well was searched, using the same strategy. Two review authors (AMB, YJ) independently screened the titles and abstracts of references identified by the electronic searches to identify publications of potentially eligible trials. These trials were assessed for inclusion in the review and discrepancies in eligibility judgements were resolved by discussion. To rate the agreement between the first and second reviewer in selecting the studies for this review, Kappa statistics was calculated. This was based on comparing the number of observed agreements with the number of agreements that would expected simply by chance, using Microsoft Excel® software. The strength of agreement is considered to be “good” when kappa values are between 0.4 and 0.75 and “excellent” when kappa ≥ 0.75. The review selection criteria were applied to each identified record and exclusion of potentially eligible studies was reported in the PRISMA diagram.

### Data extraction and management

Data from published trial reports were extracted and entered onto an electronic form (using Microsoft Excel®). Authors, year of publication, country, type of study, topical agent, severity of disease, diagnostic method, number of subjects (per protocol population), treatment regimens, placebo (if applicable), delivery system (and administrator), length of follow-up after the end of the treatment, and regression/remission rate have been tabulated.

### Quality assessment

The methodological quality of the included studies was evaluated according to Cochrane collaboration’s tool for assessing risk of bias for randomized clinical trials (RCTs) [26]. The Grading of Recommendations Assessment, Development and Evaluation (GRADE) approach was applied to assess the certainty of evidence across studies [27]. The details for these assessments will be presented in the Additional file 2.

## Results

**Fig. 1:**
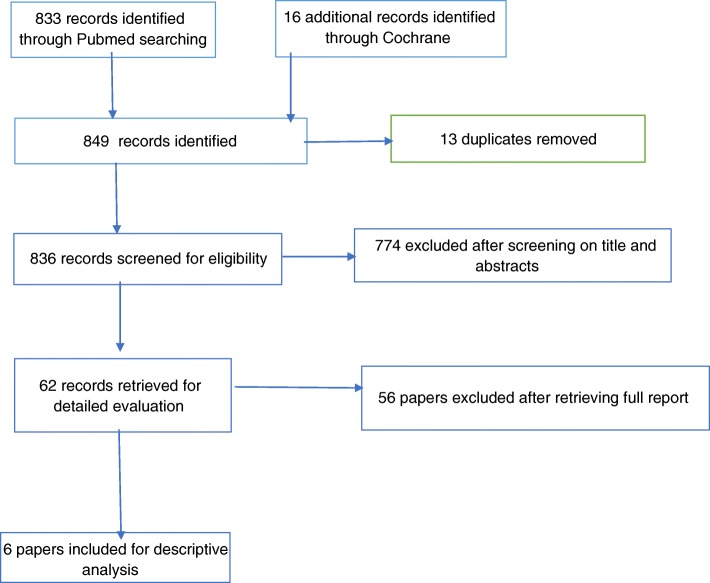
Search flow diagram

The initial search yielded 849 papers, of which 13 duplicates were removed. Out of 836 potentially relevant papers, 62 were maintained for further analysis based on the title and abstract. Six of these met the inclusion criteria and were included in the final descriptive analysis (Fig. 1: search flow diagram). Checking the reference lists of included studies and published reviews yielded no additional studies. The agreement between the reviewers in selecting these articles after applying the inclusion and exclusion criteria was analyzed with a kappa statistic, which resulted in a value of 0.90 (95% CI 0.87–0.93). The characteristics of the individual included studies are presented in Table 2. An important observation is that all the included studies originate from high-income countries. Most of them were randomized clinical trials, published between 1991 and 2014. The included studies were of varying methodology and were predominantly performed in a secondary screening setting. To highlight the risk of bias, the Cochrane risk of bias tool was utilized for the included studies. According to this, these studies were considered to be high quality (Additional file 1). Overall, the certainty of evidence across these studies was reported to be of moderate level for one intervention to low level for the remaining interventions (Additional file 2).

**Table 2:** Characteristics of the included studies

	First author, year of publication	Country	Study design	Topical agent	Diagnostic method	Number of patients (per protocol)	Disease
1	Sidhu, 1997 [14]	Ireland (UK)	RCT	5-FU	Biopsy	94	CIN 1–2
2	Rahangdale, 2014 [13]	USA	RCT	5-FU	Biopsy	55	CIN 2 (3)
3	Meyskens, 1994 [28]	USA	RCT	atRA	Biopsy	232	CIN 2–3
4	Ruffin, 2004 [29]	USA	RCT	atRA	Biopsy	174	CIN 2–3
5	Van Pachterbeke, 2009 [16]	Belgium	RCT	Cidofovir	Biopsy	48	CIN 2–3
6	Grimm, 2012 [30]	Austria	RCT	Imiquimod	Biopsy	55	CIN 2–3

*CIN* cervical intraepithelial neoplasian, *CT* clinical trial, *FU* fluorouracil, *HPV* HPV infection, *atRA* all-trans retinoic acid, *RCT* randomized clinical trial, *UK* United Kingdom, *USA* United States of America

Among the commercially available agents, the more frequently studied were 5-FluoroUracil (5-FU), Cidofovir 2%, Imiquimod 5%, and all-trans retinoic acid (atRA).

Considering clinical heterogeneity, pooling of data was not possible. Instead, a best evidence synthesis for histological, colposcopic, or cytologic regression or remission was compiled. The treatment regimen and regression/remission rates are summarized in Table 3.

**Table 3:** Overview of selected studies

	Author	Treatment regimen	Placebo	Delivery system (by investigator/patient)	Disease	Number treated/placebo	Regression treated (%)/placebo (%)	Remission treated (%)/placebo (%)	FU, after end of treatment
1	Sidhu 1997 [14]	20 mg of 5-FU for 24 h (one application)	Cytotoxic drug delivery system without 5FU	Bilaminar bioadhesive polymeric film	CIN1–2	48/46	67%/72% (*p* = 0.76)		6 months
2	Rahangdale 2014 [13]	2 g of 5% 5-FU once every 2 weeks for a total of 16 weeks (eight applications)	Observation, no placebo	Vaginal applicator (+ tampon) (by patient)	CIN2	28/27	93%/56% (*p* = 0.01)	86%/44%	10 weeks
3	Meyskens 1994 [28]	1.0 ml of 0.375% atRA or a placebo cream for 24 h. Start: daily for 4 days; months 3 and 6: daily for 2 days (eight applications)	Polyethylene glycol 400, butylated hydroxytoluene, 55% alcohol	Cervical cap + collagen sponge (by investigator)	CIN2 CIN3	75/66 40/51		43%/27% (*p* = 0.41) 25%/31% (*p* = 0.33)	21 months
4	Ruffin 2004 [29]	0.16–0.28% - 0.36% atRA or placebo daily for four consecutive days (four applications)	Same carrier base to compound atRA	cervical cap + collagen sponge (by investigator)	CIN2 CIN3	29–32–34/24 16–12–13/14	83–87–74%/70% 57–58–35%/57%	62–50–50% /62% 44–50–14/21%	11 weeks
5	Van Pachterbeke 2009 [16]	3 ml cidofovir 2% in Intrasite gel (for 4 h) or placebo, three applications in 1 week	Sterile water + Intrasite gel	Cervical cap (by investigator)	CIN2–3	23/25		61%/20% (*p* = 0.01)	6 weeks
6	Grimm 2012 [30]	6.25 mg imiquimod (overnight)	2 g Adeps solidus	Vaginal suppositories (by patient)	CIN2–3	28/27	79%/41% (*p* = 0.009)	50%/15% (*p* = 0.08)	4 weeks
		week 1–2: 1 suppo/w							
		week 3–4: 2 suppo/w							
		week 5–16: 3 suppo/w							
		(max. 42 applications)							

*CIN* cervical intraepithelial neoplasia, *FU* follow-up, *mIU* million international units

Two studies involved topical 5-FU in 149 CIN1–2 women [13, 14]. In the study by Sidhu et al., the regression rate was 67% in the 5-FU-treated group compared to 72% in the placebo group (*p* = 0.76) (no remission data available) [14]. However, Rahangdale et al. reported regression rates of 93% (86% remission) in the 5-FU-treated group compared to 56% (44% remission) in the control group (= observation) (*p* = 0.01) [13]. The treatment schedule, delivery system, and follow-up time completely differed between the two studies.

Another two papers [28, 29] reported on the efficacy of trans-retinoic acid treatment in 406 women with lesions. Both studies showed comparable results for equal concentrations of atRA (0.375% and 0.36%), although the number of applications and follow-up time were very diverse. The remission rates were 43% and 50% for CIN2 lesions, and 25% and 14% for CIN3 patients, in the Meyskens and Ruffin study respectively. A huge difference in remission could be noticed between the placebo controls in both studies (24% remission (15) versus 62% (16) for CIN2 lesions), and atRA was therefore judged no more effective than placebo in the Ruffin study. Lower concentrations of atRA (0.16–0.28%) showed better remission in the CIN3 (44–50%) study group compared to the 0.36% of atRA (16).

In small study set ups (48 and 55 women, respectively), the reported remission of grouped CIN2–3 cases was 61% for Cidofovir (versus 20% for placebo) (*p* = 0.01) and 50% for Imiquimod (versus 15% for placebo) (*p* = 0.08) [16, 30].

No moderate or severe side effects related to drug or placebo exposure were reported, except in the study by Meyskens et al. in 1994, where mild and reversible vaginal and vulvar side effects were seen in the patients receiving retinoic acid [28]. An overview on the studies which were excluded after full-text review and reasons for exclusion are presented in Table 4. Most of the excluded papers were about what is called photodynamic therapy which does not act as a biological agent [31–46].

**Table 4:** Reasons for exclusion of studies

Reasons for exclusion	Studies
In vitro study on cervical cancer lines	Zou 2003
Less than 20 women involved	Barten1987; Pride 1982; Choo1986; Hubert 2012; Snoeck 2000; Choo 1985
Paper not traceable (no abstract available)	Marianowski 1976; Misiewicz 1969
Phase I clinical trial	Meyskens 1986; Weiner 1986; Cheng 2001
Photodynamic therapy	Barnett 2003; Choi 2013; Choi 2014; Fu 2016; Hillemanns 2015, Hillemanns 1999; Hillemanns 2014; Hillemanns 2008; Hillemanns 2015; Paheernik1998; Soergel 2010; Yamaguchi 2005; Wang 2010; Monk 1997; Soergel 2008
Restrospective case series	Geisler 2016
Review, and/or not a randomized clinical trial	Cinel 1991; Snoeck 2001; Chakalova 2004; Tao 2014
Surgical treatment utilized	Pachman 2012; Koeneman 2016; Maiman 1999; Gleeson 1992
Vulvar and/or vaginal intraepithelial neoplasia also treated	Diaz-Arrastia 2001; Lin 2012; Yliskoski 1990
Study on cervical condylomatosis	Alberico 1985
Study on HPV infection only	Shukla 2009; Iljazovic 2006; Niwa 2003; Chen 2013; Cappello 1991; Schettino 2013
Systemic route also utilized	Rotola 1995; Valencia 2011; Follen 2001; Vlastos 2005; Sartor 1993; Osnengo 1990; Alvarez 2016

## Discussion

During the last two decades, topical biological agents have been studied as alternatives to surgical methods for the management of cervical intraepithelial neoplasia (CIN). The objective of this review was to report on the efficacy of commercially available topical drugs used in the treatment of CIN. A total of six studies fulfilling all the inclusion criteria were retained for this review. They reported on 5-FluoroUracil [13, 14], all-trans retinoic acid [28, 29], Imiquimod [30], and Cidofovir [16]. Although this number was relatively low, our inclusion criteria focused on the commercially available drugs and on randomized clinical studies. The reported agents in the present review have been shown efficacious in clinical regression of CIN. Regression rates, however, differ among studies. One of the reasons for that is their varying methodological design and therapeutic schemes. Considering the results in their globality, we strive to find that each of the agents does produce regression or remission regardless of the regimen or the method employed for diagnosis or follow-up. In general, remission ranged between 43 and 93% for the active agents in CIN2 patients.

5-Fluorouracil is a pyrimidine analogue which acts through inhibition of the target enzyme thymidylate synthase (TS) by the 5-FU metabolite, FdUMP, and incorporation of this metabolite into DNA, resulting in inhibition of DNA synthesis and function. It stops the cells making and repairing DNA. Topical 5-FU appears to be an effective medical therapy for CIN [13, 14]. Although 5-FluoroUracil resulted in 93% regression versus 56% for placebo in the study by Rahangdale et al. [13], Sidhu et al. [14] found a regression of 67% for 5-FluoroUracil less efficacious compared to placebo (72%). The latest was a double-blind randomized controlled trial while the study by Rahangdale et al. was a prospective but nonblinded randomized trial of 5-FU versus standard-of-care observation (no placebo). The difference in the methodology and the regimen (one versus eight applications) can explain the discrepant results between these studies. Although Rahangdale et al. deliberately did not use a placebo to avoid interfering with the natural regression of CIN, this has constituted a limitation. Furthermore, this study introduced bias since the investigator, in a clinical setting, was not blind to the randomization allocation and could have been influenced in performing colposcopically directed biopsies, resulting in wrong diagnoses. The high spontaneous regression rate of CIN in the placebo group found by Sidhu et al. and Rahangdale et al. can be explained by the fact that patients with CIN3 lesions were excluded from these studies, thus allowing background spontaneous regression to be a confounding variable. Further on, the cytotoxic drug delivery system used in the Sidhu trial could have altered the pH at the cervical epithelium and this may have been sufficient to cause cell death, resulting in the clearance of the lesion.

Retinoids are essential for cell growth, differentiation, and cell death. Various retinoids have been shown to inhibit cellular proliferation in cervical cancer cells in several studies. All-trans retinoic acid (atRA) is an active metabolite of vitamin A under the family retinoid. Retinoids, through their cognate nuclear receptors, exert potent effects on cell growth, differentiation, and apoptosis. Retinoic acid either decreases or increases EGF-stimulated growth and epidermal growth factor-receptor (EGF-R) expression depending on the cell line and culture conditions. HPV-containing cell lines overexpress EGF-R and are more sensitive to retinoids than normal cells. Increased sensitivity of HPV-containing cells may explain the reversal of premalignant lesions and dysplasias of the cervix by retinoic acid [22]. In a study on all-trans retinoic acid by Ruffin et al. [29], the rate of histologic remission in biopsied CIN II patients was 50% (0.36% atRA) over a short treatment time of 4 days (FU time = 11 weeks), but no difference with the remission rate in the placebo controls (62%) could be observed, judging the atRA treatment as no more effective than placebo treatment. In the study by Meykens et al. [28], a comparable remission of 43% (0.375% atRA) was obtained with a double amount of applications and a much longer follow up time (21 months). It was suggested in this study that locally applied RA could favorably alter the natural history of CIN if the lesion had not progressed too far (no difference between RA and placebo in CIN3 patients). The authors hypothesized that this significant regression rate—compared to their placebo control (only 27% remission)—could be explained by the fact that RA has been an effective inhibitor of growth and stimulator of differentiation in cervical tissue by producing immunologic and inflammatory responses. An important constatation is that lower concentrations of atRA (0.16% and 0.28%) gave better results in the CIN3 patient group (44% and 50% regression, respectively, compared to 14% for the higher atRA concentration of 0.36%) [28].

For some agents, only one study could be retrieved for this systematic review. Imiquimod is an immunomodulator with antiviral and anti-tumor effects. It is a toll-like receptor 7 agonist and induces upregulation of interferon and activation of dendritic cells. Imiquimod (5% cream) has been shown to be safe and effective in the treatment of genital warts caused by low-risk HPV infections. The mechanism for the eradication of genital verrucous lesions with imiquimod may involve the induction of both innate and cellular immunity. Antiviral activity may be stimulated through the induction of cytokines, such as interferon-a (IFN-a), tumor necrosis factor-a (TNF-a), and interleukins (ILs) [18, 19]. In a randomized, double-blind, placebo-controlled phase II trial of Imiquimod, the histologic remission of 50% was significantly higher than the 15% in the placebo-controls. Topical, patient-applied, vaginal imiquimod therapy was therefore demonstrated to be an efficacious treatment option for CIN2–3 patients in this study. The most important shortcoming was the very short follow-up after the completion of the full treatment (4 weeks), warranting a study with a longer follow-up period [30]. A new study is on its way, whose purposes are to test the treatment efficacy hypothesis as well as long-term disease recurrence after treatment and clinical applicability of imiquimod treatment, and to develop a prediction model for clinical response to Imiquimod treatment, based on histological biomarkers [47].

Cidofovir, an acyclic phosphonate nucleoside with a broad-spectrum activity against DNA viruses, has proved efficacious in the treatment of different clinical manifestations of HPV-induced epithelial cell proliferation. As an antiviral drug, it is incorporated in viral DNA and preferentially inhibits DNA, reduce capacity of HPV positive cells to repair DNA damage. It is recognized as one of the effective treatments for several HPV-induced lesions [17, 48]. Cidofovir 1% gel is able to inhibit partially or completely cervical dysplasia lesions [16, 17] . In a study by Van Pachterbeke et al., cidofovir was shown to be active against CIN2^+^ lesions in immunocompetent women. In this study, histological clearance was demonstrated in 61% compared with 20% in the placebo group. The failure to regress completely may be due to insufficient penetration to all targeted cells, to the frequency of administration of the product during the study, or to the severity of the lesion. The short observation time of 6 weeks was again an important weakness. Most of the studies analyzing the data after a longer period (3 to 6 months) provided more complete responses [16]. Thus, it has to be verified whether a prolonged exposure to topical drugs could provide better outcomes. The last two studies grouped the results of CIN2 and CIN3 patients together, so higher remission rates can be expected in an exclusively CIN2 population with Imiquimod and Cidofovir, based on the varying remissions according to the grade of CIN in the studies with separate CIN2 and CIN3 data [16, 30].

Although the six studies included in this systematic review provided evidence to allow conclusions to be drawn, the review has several limitations. The treatment doses and regimen and the length of follow-up varied among studies. This also made it difficult to pool the results for the included studies. Differences in populations and stages of CIN made comparisons among studies challenging. Therefore, further large-scale, rigorous RCTs with consistent inclusion criteria, design, and outcomes are warranted to ascertain the efficiency of the different treatment approaches.

To conclude, regression results for the reported topical agents were discrepant between studies. 5-FluoroUracil has provided good results in term of CIN remission. Moreover, the latter is known to be a very active anticancer drug used in chemotherapy for various types of invasive neoplasms. In the framework of secondary prevention, topical therapies can play an important role as they may help in safely treating precancerous lesions without resorting to surgical methods. This is a great advantage because surgical methods may have many complications in the short and long term. Another advantage of the topical drugs reviewed here is their ability to be stored at room temperature, thus not requiring cold chain, as it is known that in developing countries, the cold chain may be interrupted due to non-availability of electrical current. This allows their proper use in low- and middle-income countries (LMICs) when they need to come from abroad or when they should be stored for a long time in rural areas.

## Conclusion

The efficacy of topical agents in the studies reviewed were variable depending on the agent and methodology employed. Cidofovir, Imiquimod, all-trans retinoic acid, and 5-FluoroUracil have been proposed as agents for topical treatment of CIN lesions. Overall, the remission rates of CIN2 ranged from 43 to 93%. However, sometimes results have been contradictory for a given agent. One of the explanations is that the methodology and the regimen employed are different from one study to another. Hence, to shed light on the true efficacy of these topical agents, large-scale, multicentric, and less biased studies are necessary. When proved effective, the topical agents could advantageously be used as an alternative to surgery in treating HPV-associated precancerous lesions of the cervix, especially for the LMICs.

## Additional files

Additional file 1: Cochrane risk of bias tool for randomized controlled trials. (DOCX 17 kb)

Additional file 2: GRADE Table for Certainty of Evidence. (DOCX 20 kb)

## Abbreviations

5-FU: 5-Fluorouracil
atRA: All-trans retinoic acid
CC: Cervical cancer
CIN: Cervical intraepithelial neoplasia
CT: Clinical trial
DNA: Deoxyribonucleic acid
EGF-R: Epidermal growth factor-receptor
HPV: Human papillomavirus
IFNs: Interferons
ITT: Intention to treat
LEEP: Loop electroexcision procedure
LMICs: Low- and middle-income countries
mIU: Million international units
RCT: Randomized clinical trial
RNA: Ribonucleic acid
TBS: The Bethesda System
UK: United Kingdom
USA: United States of America
VIA: Visual inspection with acetic acid
