# Supplementary material for: Efficacy of commercially available biological agents for the topical treatment of cervical intraepithelial neoplasia: a systematic review
Source: Syst Rev. 2019 Jun 7;8:132. doi: 10.1186/s13643-019-1050-4 (PMC6555029; doi:10.1186/s13643-019-1050-4)
Supplement: Supplementary file 1 — Cochrane risk of bias tool for randomized controlled trials. (DOCX 17 kb) [file 13643_2019_1050_MOESM1_ESM.docx]

Additional file 1: Cochrane risk of bias tool for randomized controlled trials

| **Study**  **Author** | **Year** | **Was randomization adequate?** | **Was allocation concealment adequate?** | **Were care providers masked?** | **Were patients masked?** | **Were outcome assessors masked?** | **Was overall attrition ≥20%?** | **Did the study use ITT analyses?** | **Selective reporting?** | **Other bias?** | **Risk of Bias** |
| --- | --- | --- | --- | --- | --- | --- | --- | --- | --- | --- | --- |
| Sidhu | 1997 | Yes | Yes | Yes | Yes | Yes | No | Yes | No | No | Low |
| Rahangdale | 2014 | Yes | Yes | No | No | Yes | No | Yes | No | No | Low |
| Meyskens | 1994 | Yes | Yes | Yes | Yes | Yes | Yes | Yes | No | No | Low |
| Ruffin | 2004 | Yes | Yes | Yes | Yes | Yes | No | Yes | No | No | Low |
| Van Pachterbeke | 2009 | Yes | Yes | Yes | Yes | Yes | No | Yes | No | No | Low |
| Grimm | 2012 | Yes | Yes | Yes | Yes | Yes | No | Yes | No | No | Low |

ITT = Intention-to-treat analysis
