# Supplementary material for: Efficacy of commercially available biological agents for the topical treatment of cervical intraepithelial neoplasia: a systematic review
Source: Syst Rev. 2019 Jun 7;8:132. doi: 10.1186/s13643-019-1050-4 (PMC6555029; doi:10.1186/s13643-019-1050-4)
Supplement: Supplementary file 2 — GRADE Table for Certainty of Evidence. (DOCX 20 kb) [file 13643_2019_1050_MOESM2_ESM.docx]

Additional file 2: GRADE Table for Certainty of Evidence

| **Quality assessment** | | | | | | | | **No of patients** | **Overall certainty of evidence** |
| --- | --- | --- | --- | --- | --- | --- | --- | --- | --- |
| **Intervention/Outcome** | **No of studies** | **Design** | **Limitations** | **Inconsistency** | **Indirectness** | **Imprecision** | **Publication bias** |  |  |
| **CIN Regression** | | | | | | | | | |
| 5-Fluorouracil | 2 | RCT | Serious^a^ | Serious^b^ | No serious indirectness | No serious imprecision | None | 149 | Low |
| all-trans retinoic acid | 2 | RCT | Serious^c^ | No serious inconsistency | No serious indirectness | No serious imprecision | None | 406 | Moderate |
| Imiquimod | 1 | RCT | No serious limitations | No serious inconsistency | No serious indirectness | Very serious^d^ | None | 55 | Low |
| **CIN Remission** | | | | | | | | | |
| Cidofovir | 1 | RCT | No serious limitations | No serious inconsistency | No serious indirectness | Very Serious^d^ | None | 48 | Low |

Footnotes

Abbreviation: RCT, randomized controlled trial; CIN, cervical intraepithelial neoplasia

^a^ Inadequate allocation concealment, unblinding of participants and providers in one study (Rahangdale)

^b^ unexplained herogeneity of results, success rate did not differ significantly between drug group and placebo group in one study (Sidhu)

^c^ high dropout rate in one study (Meyskens)

^d^ includes relatively very few patients
